# Respiratory Muscle Dysfunction and Associated Risk Factors Following COVID-19-Related Hospitalisation

**DOI:** 10.3390/life15020194

**Published:** 2025-01-28

**Authors:** Alessia Verduri, Roberto Tonelli, Pierluigi Donatelli, Jonathan Hewitt, Giovanni Guaraldi, Jovana Milić, Valentina Ruggieri, Cristina Mussini, Enrico Clini, Bianca Beghè

**Affiliations:** 1 Respiratory Unit, Hospital Policlinico Modena, Department of Surgical and Medical Sciences, University of Modena and Reggio Emilia, 41125 Modena, Italyvalentina.ruggieri@unimore.it (V.R.); enrico.clini@unimore.it (E.C.); bianca.beghe@unimore.it (B.B.); 2 Department of Population Medicine, Cardiff University, Cardiff CF24 4AG, UK; hewittj2@cardiff.ac.uk; 3 Infectious Diseases Unit, Hospital Policlinico Modena, Department of Surgical and Medical Sciences, University of Modena and Reggio Emilia, 41125 Modena, Italy; giovanni.guaraldi@unimore.it (G.G.); jovana.milic@gmail.com (J.M.); cristina.mussini@unimore.it (C.M.)

**Keywords:** post-COVID, maximal inspiratory pressure (MIP), maximal expiratory pressure (MEP), hand-grip strength, lung function, non-invasive ventilation

## Abstract

Background: Studies have highlighted long-term respiratory muscle dysfunction in COVID-19 survivors, although the underlying risk factors remain unclear. This single-centre study assessed respiratory muscle function and individual associated factors at follow-up in patients hospitalised with COVID-19 and related acute respiratory failure. Methods: Data were collected for consecutive patients, aged ≥ 18 years, at the post-COVID outpatient service of Hospital Policlinico in Modena (Italy) in the time frame of 3 to 6 months after discharge. Data were analysed using single and multiple logistic regression models. Correlations among MIP/MEP, hand-grip values, and lung function were further explored. Results: Out of 223 patients (mean age 67 years, 69% male) 121 (54.3%) exhibited MIP or MEP dysfunction, which was found to be associated with the use of non-invasive ventilation (aOR = 1.91 [1.07–3.49], *p* = 0.04) and female gender (aOR = 1.76 [1.09–4.16], *p* = 0.03) as independent risk factors. A positive correlation was observed between MIP dysfunction and hand-grip strength (*p* = 0.03 and 0.01), whereas both MIP and MEP were significantly associated with FEV_1_, FVC, TLC, and DLCO. Conclusions: Respiratory muscle dysfunction is consistently prevalent and parallels peripheral muscle weakness and the lung function level in patients at follow-up after severe COVID-19. The need for non-invasive ventilation during the acute phase and female gender might represent risk factors. MIP/MEP assessment should be recommended to observe respiratory muscle dysfunction in severe post-COVID survivors.

## 1. Introduction

The Coronavirus disease 2019 (COVID-19) pandemic was declared on 11 March 2020 from the World Health Organization (WHO) and had an unprecedented impact on global health in terms of morbidity and mortality. Beyond the 7,065,880 deaths confirmed in September 2024 [1], it is known that the symptoms of COVID-19 can range from asymptomatic to life threatening [2]. People experience a wide range of symptoms, including fever, dry cough, shortness of breath, muscle pain, and fatigue, although most infections are not severe, and people are able to recover at home without hospital treatment. Where the novel Coronavirus causes severe illness, the most common clinical presentation is bilateral pneumonia leading to hypoxemia with acute respiratory failure. Individuals developing moderate-to-severe respiratory failure require hospital admission with the need for respiratory support, such as oxygen therapy and/or non-invasive ventilation (NIV).

A significant proportion of patients post COVID-19, particularly those with severe acute disease, may experience persistent symptoms with a poor quality of life following recovery from the viral infection. Increasing evidence of the post-COVID long-term effects following hospitalisation has been reported in literature. Clusters of symptoms, including dyspnoea, fatigue, muscle pain, and sleep disturbances have been frequently described and indicated new disability [3,4].

Considering that SARS-CoV-2 is a virus spreading to multiple organs other than the lungs, it has been proposed that the COVID-related cytokine storm may cause dysfunction to the respiratory muscles (RMs), especially the diaphragm [5,6]. On the other hand, critical illness myopathy is associated with prolonged mechanical ventilation and patient immobilisation, which are risk factors for complications, such as peripheral muscle weakness and RM dysfunction [7,8]. Skeletal muscle is one of the most involved organs in COVID-19. Myalgia and fatigue are the third-most reported symptoms (after fever and cough) in people with symptomatic infection [9].

The relationship between COVID-19 and changes in RM and the resulting functional implications have not been yet completely understood in individuals without pre-existing conditions associated with RM dysfunction. In this study, we aimed to explore the following: (1) long-term RM functions in patients previously hospitalised for COVID-19; (2) factors associated with RM dysfunction, and the association of MIP/MEP levels with the follow up variables.

## 2. Materials and Methods

### 2.1. Study Design

We established a post-COVID follow-up service at the Outpatient Department of Hospital Policlinico in Modena (Italy), for patients admitted with COVID-19 since the first wave of the pandemic. The primary setting that provided patient assessments was a multidisciplinary referral centre for post-acute COVID syndrome at the Infectious Disease Unit and, secondly, at the Respiratory Unit. The study was granted by the Ethics Committee of the Azienda Ospedaliera-Universitaria Modena (453/2020/OSS/AOUMO-CoV-2 MO-Study).

We aimed to follow up all patients in-person after discharge and collected data also to identify people presenting with any persistent symptom. This study is an observational single-centre cohort study that has been designed and carried out on those patients who attended the service solely 3 to 6 (three-to-six) months after discharge. All visits were conducted in an optimal time window.

### 2.2. Participants

All consecutive patients referred to the post-COVID service between June 2020 and October 2021, aged ≥ 18 years, with a minimum 7-day length of hospital stay, were systematically included. Exclusion criteria were a pre-admission diagnosis of chronic conditions potentially affecting RM functions, such as asthma, COPD, ILD, coronary artery disease and/or chronic heart failure, cardiomyopathies, and neuromuscular diseases. Nursing home residents were excluded due to the difficulty of attending follow-up appointments. Patients not able to perform lung function tests and hand-grip tests were also excluded.

### 2.3. Procedures

At the follow-up visit, patients were subjected to spirometry with diffusing capacity for carbon monoxide (DLCO) [10,11], measurements of the maximal -inspiratory -pressure (MIP) and maximal expiratory pressure (MEP) [12,13], hand-grip test [14], and the modified Medical Research Council (mMRC) dyspnoea scale [15].

### 2.4. Variables and Outcomes

#### 2.4.1. Variables

Variables collected at follow up included the following: age; sex; smoking status (never, previous, or current); pre-admission body mass index (BMI); respiratory support due to severe hypoxemia (non-invasive ventilation (NIV); high-flow nasal cannula (HFNC) oxygen therapy; intubation); use of Dexamethasone; use of any other systemic corticosteroid; use of Tocilizumab; O_2_ at discharge; length of stay in hospital (LOS) defined as the entire duration of hospitalisation, including the length of stay in Intensive Care Unit (ICU); time from discharge to follow-up; pulmonary function parameters, DLCO, MIP, and MEP; skeletal muscle strength of the hand; and dyspnoea scale. Data collection was completed using patient clinical records (case notes and electronic records) and drug prescription charts. All data were entered into a specific database.

#### 2.4.2. Outcomes

The primary aim was to explore the prevalence of maximal inspiratory pressure (MIP) and maximal expiratory pressure (MEP) dysfunction in COVID-19 survivors, according to the age-adjusted lower threshold from Lista-Paz, 2023 [13]. The secondary outcomes were as follows: (1) MIP/MEP dysfunction related risk factors; (2) association of MIP/MEP levels with the follow up variables. The peripheral muscle dysfunction was defined as a reduction in the dominant-hand grip strength under the 25th percentile (age- and sex-adjusted value) [14]. The lung volume reduction was defined either as a restrictive pattern (TLC < 90% of predicted value) or reduced lung diffusion capacity (DLco < 80% of predicted value). A self-reported symptom of dyspnoea according to an mMRC scale score ≥ 1 indicated the presence of breathlessness [15]. Outcomes were assessed up to the last data entry using systems of prospective follow-up and electronic health records.

### 2.5. Data Analysis

A priori sample size calculation on the primary outcome was based on the reported prevalence of MIP/MEP dysfunction among critically ill patients ranging as 50–80% [16,17,18]. Assuming α = 0.05 and power of 85 and a margin of error of 5%, a sample size of 223 patients was suitable for assessing the primary outcome. Data were displayed as the median and interquartile range (IQR) for continuous variables and numbers and percentages for dichotomous variables. Continuous variables were compared using a Student’s *t*-test or Mann–Whitney test as appropriate. Categorical variables were expressed as numbers and percentages (%) and compared using a χ2 test or Fisher’s exact test across the groups. The association with MIP/MEP dysfunction was analysed using a univariate single logistic regression model and a multiple logistic regression model to explore the independent associations for selected variables. Sensitivity analysis was used to examine the correlation between the values of MIP and MEP (expressed as % of predicted values) and hand-grip values and pulmonary function parameters (namely TLC, FEV_1_, FVC, and DLCO). Pearson’s R or the Spearman correlation coefficient were used as appropriate. Statistical analysis was performed using SPSS package ver.25.0 (IBM Corp., Armonk, NY, USA) and GraphPad Prism version 8.0 (GraphPad Software, Inc., La Jolla, CA, USA) unless otherwise indicated.

## 3. Results

Between June 2020 and October 2021, 719 participants with COVID-19 were assessed at the follow-up service. Of these, 496 were excluded based on the study criteria, missing data, and the follow-up time not ranging within 3–6 months. The study included 223 patients. The time from discharge to follow-up was 4 ± 1.1 months (mean ± SD). Demographic and clinical variables are shown in Table 1. Overall, the mean age was 67 years with a greater proportion of men (69%). The mean BMI was 30. On admission, conventional oxygen therapy (COT) was used for 24%, HFNC oxygen therapy for 27%, and NIV for 33%. The intubation rate was 16% (36/223). Missing data accounted for less than 3% of the dataset.

### 3.1. Prevalence of MIP/MEP Dysfunction and Related Risk Factors

A reduction in the MIP or MEP was found in 54.2% of patients (Table 1). In the multiple logistic regression model, MIP or MEP dysfunction showed positive associations with the use of NIV (*p* = 0.04) and the female gender (*p* = 0.03) (Table 2).

### 3.2. Correlation Among MIP/MEP, Peripheral Muscle Function, Lung Function, and Dyspnoea

**Figure 1:**
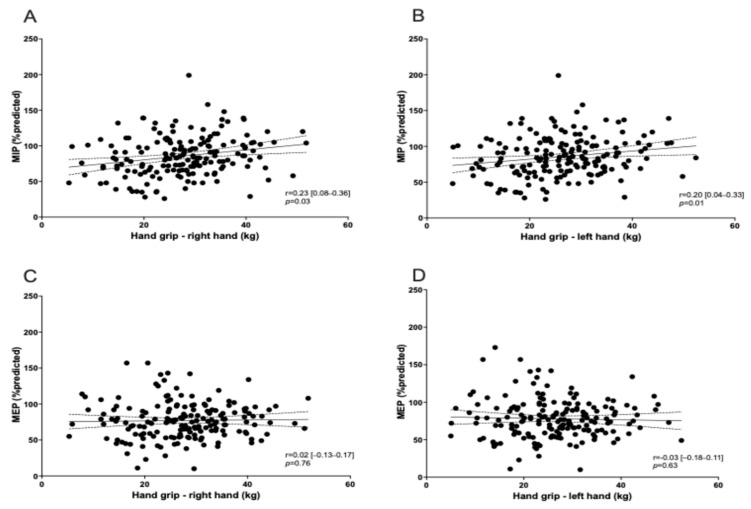
Correlation between MIP/MEP and hand-grip strength. Correlation is shown for MIP with right hand grip (**A**) and left hand grip (**B**), as well as for MEP with right hand grip (**C**) and left hand grip (**D**).

**Figure 2:**
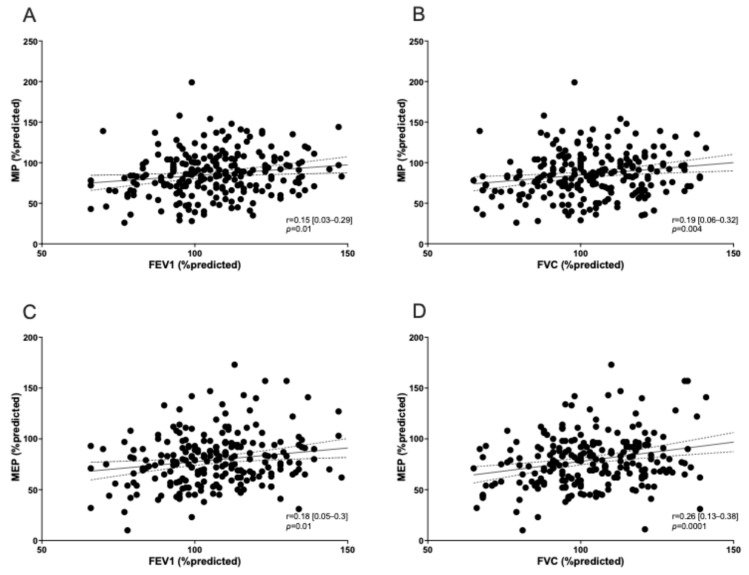
Correlation among MIP/MEP and FEV_1_ and FVC values. Correlation is shown for MIP with FEV_1_ (**A**) and FVC (**B**), as well as for MEP with FEV_1_ (**C**) and FVC (**D**).

**Figure 3:**
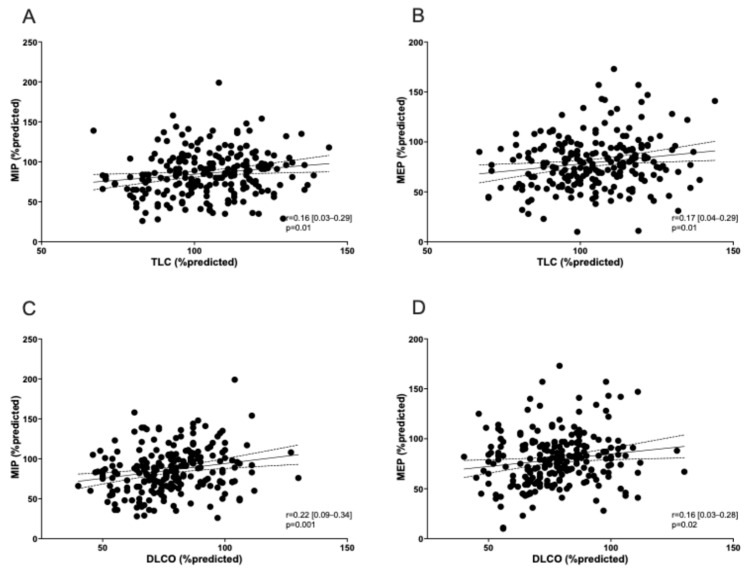
Correlation among MIP/MEP and TLC and DLCO values. Correlation is shown for MIP with TLC (**A**) and DLCO (**C**), as well as for MEP with TLC (**B**) and DLCO (**D**).

A reduction in dominant hand grip strength (in kg) was observed in 27% of patients (Table 1). Using the Pearson correlation coefficient test, right and left hand grip strength was associated with MIP dysfunction (*p* = 0.03 and 0.01, respectively) (Figure 1). None of the patients showed an obstructive pattern on spirometry (FEV_1_/FVC < 0.7). A restrictive pattern (TLC < 90% pred) was observed in 17% of patients and reduced lung diffusing capacity (DLCO < 80% pred) was found in 57.4% (128 participants). All patients presenting a restrictive pattern on spirometry showed a reduction in DLCO. MIP and MEP were significantly correlated with the lung function level (Figure 2) and DL_CO_ (Figure 3). mMRC ≥ 1 grade was reported in 67/223 patients (30%) (Table 1), and no association was found with RM dysfunction.

## 4. Discussion

In this cohort study of 223 patients hospitalised with severe COVID-19, we found MIP or MEP dysfunction in 54.2% of participants at follow-up ranging from 3 to 6 months, excluding those with pre-existing pulmonary, cardiac, and neuromuscular comorbidities.

The study adds to the evidence base evaluating RM functions in COVID-19 survivors [19,20,21]. One previous study found a reduction in the MIP and MEP in 49.1% and 22.8%, respectively, among slightly younger patients at 1 month from hospital discharge [19], and no correlation was observed with the use of corticosteroids or severe COVID. That population was characterised by 30% of severe cases without underlying pulmonary and cardiovascular disease. Correspondingly, we also did not find any association with glucocorticoid therapy and/or illness severity or intubation. Another study demonstrated a greater decrease in MIP (27%) than MEP at the same timepoint in patients who presented higher intubation rates (40%) and pre-admission lung disease (29%) [20]. A multicentric study showed an MIP reduction even 30 months following hospitalisation for severe COVID-19 in younger patients, after excluding chronic diseases that lead to RM dysfunction [21]. Whilst the authors did not report data on intubation or mechanical ventilation, these findings were associated with a prolonged hospital stay and age. Our findings are consistent with previous data on medium- and long-term outcomes regarding RM dysfunction in individuals exposed to severe COVID-19 but without underlying RM-affecting diseases.

As MIP has a strong relationship with the strength of the diaphragm, which is the major inspiratory muscle, a few studies also included diaphragm ultrasound measurements following ICU admission due to COVID-19. One study, considering the predicted inspiratory muscle normal threshold level as MIP > 70%, confirmed that mechanically ventilated COVID-19 survivors had overall dysfunction at 3 (48%) and 6 (24%) months but not any specific diaphragm dysfunction [8]. In contrast, one complex study demonstrated a diaphragm dysfunction in 80% of patients not requiring mechanical ventilation, at 15 months [22]. Another study showed a significant reduction in diaphragm contractility in COVID-19 survivors [23]. The persistence of MIP reductions in our population highlights the substantial impact of severe COVID-19 on inspiratory muscle functions, even in the absence of prolonged mechanical ventilation or other established contributors to RM impairment.

This impairment is likely to be linked to a combination of factors, including systemic inflammation, microvascular damage, and critical illness myopathy. Additionally, mechanisms, such as ventilator-induced diaphragm dysfunction and oxidative stress, may further impair contractility and strength. Unlike MEP, MIP appears particularly vulnerable due to the diaphragm’s central role in respiration and its heightened sensitivity to systemic and illness-related factors.

We also found that MIP/MEP dysfunction was associated with the use of NIV as an independent risk factor. Prolonged NIV use may contribute to diaphragmatic deconditioning, promoting long-term impairment as a result. Interestingly, the number of patients with reduced MIP or MEP exceeded those requiring NIV (33%) or intubation (16%). Whilst ICU admission and mechanical ventilation, which can lead to critical illness myopathy [8,24], may explain RM dysfunction in some severe COVID-19 cases, the persistence of an MIP reduction in non-hospitalised patients or those requiring no respiratory support, as observed in our study, requires further investigation. Evidence of RM dysfunction in survivors, regardless of the mechanical ventilation status, remains inconclusive. Factors, such as ageing and its associated decline in respiratory muscle performance, may also play a role [25].

In our study, mechanical ventilation was not associated with RM dysfunction, whereas NIV was—a difference that may be attributed to survivorship bias. Many intubated patients did not survive, excluding the most severe cases from follow-up analysis, whereas, in contrast, NIV patients, unless under a therapeutic ceiling, were more likely to survive and be included. This overrepresentation of NIV patients amplifies its apparent statistical association with RM dysfunction, underscoring the need for the cautious interpretation of these findings.

We also found that the female gender was an independent risk factor for reduced MIP or MEP. This finding is in line with recent literature showing that females had a marked reduction in MIP [21], even in women not admitted to the hospital for COVID at 5 months following discharge [26]. Although the role of gender should be further clarified, Hennigs et al. [26] and Steinbeis et al. [27] demonstrated that all patients with RM dysfunction were also symptomatic at follow-up. An RM reduction was associated with dyspnoea and fatigue after 3–8 months, irrespective of hospitalisation, admission to the ICU, or a need for oxygen [27]. Nonetheless, when we assessed breathlessness, the prevalence of an mMRC score ≥ 1 (as clinically symptom presence) was 30% and not related to RM dysfunction. These findings of dyspnoea are consistent with the literature on large populations of post-COVID patients, showing a prevalence ranging from 20% to 43% in the time frame of 2–8 months follow up [28,29,30,31,32].

We also found that hand-grip strength was lower in 27% of patients, similarly to the study of Johnsen et al. (28%) who reported a prevalence of 28% at 3 months, regardless of hospitalisation, though a higher prevalence among admitted patients (32%) was reported [33]. In contrast, Godoy et al. observed only a 14% reduction in hand-grip strength at 4 months [34], while Hussain et al. reported a prevalence of 24% among ICU-admitted patients after 1 year [35]. Unlike these studies, our cohort excluded patients with relevant comorbidities. Although we cannot confirm normal hand-grip strength prior to hospitalisation, the observed changes in peripheral muscle strength were likely due to a SARS-CoV-2 infection. In addition, both right and left hand-grip strength were associated with MIP dysfunction in our cohort, suggesting a relationship between peripheral and respiratory muscle function. Whilst the lack of an association between MEP and skeletal muscle strength might seem controversial, our findings suggest that peripheral muscle function and RM function are related each other, which is in line with data reporting parallel dysfunction in older healthy individuals [36]. In addition, one previous study showed that both older age and obesity were linked to a low muscle quality index—including hand-grip strength as a component—and a concomitant reduction in MIP/MEP at 3 months after admission for COVID-19 [37]. These data further support the interrelationship between peripheral and respiratory muscle dysfunction in post-COVID cases.

We also found a mild reduction in DLCO in 57% of patients. This was expected as potential residual lung damage following extensively acute lung involvement. Our findings are in line with previous studies. A systematic review confirmed that reduced DL_CO_ was the most frequent residual functional abnormality with a prevalence of 39% at 6 months after recovery [38]. One study showed a higher prevalence of DL_CO_ reductions (76.5%) in the 30-day follow-up [19], whereas another reported comparable rates to our findings in patients treated with HFNC, NIV, or intubation at 6 months. In contrast, a lower prevalence (29%) was found in patients requiring only COT [31]. We also observed that DL_CO_ was reduced in patients showing a restrictive pattern on spirometry (17%), in line with the literature data [27]. Additionally, MIP/MEP values were globally associated with the lung function parameters (FEV_1_, FVC, TLC, and DL_CO_) at follow-up. However, the impact of these findings in people without chronic diseases potentially affecting pulmonary functions remains poorly understood. Generally, published research on COVID-19 survivors showed that DL_CO_ is the most accurate respiratory functional parameter [19,27,31,38,39,40], which should be monitored in the post-COVID population.

It is worth noting that our study was single-centre. The population included is reflective of the wider post-COVID populations during the first waves of the pandemic [2,41] but might not reflect those of different contexts.

Further limitations of note were that we did not collect imaging data examining the extension of lung abnormalities, such as a chest CT that could explore the relationship with functional data, and real-time diaphragm ultrasound in patients with MIP dysfunction. Whilst exploring the relationship between imaging data and functional data would have been interesting, it is beyond the scope of this article. Nonetheless, excluding patients with pre-existing respiratory/cardiac or neuromuscular diseases helped to better evaluate the impact of the viral pneumonia on RM functions, lung function, and dyspnoea in the absence of potential confounding factors.

Another limitation is that, whilst patients’ health care records did not report the presence of pulmonary hypertension, this should be explored by echocardiography/right heart cathetherisation data that were lacking in most patients in the first waves of the pandemic.

## 5. Conclusions

The study assessed RM strength in COVID-19 survivors and showed that most people had RM dysfunction at follow-up. In addition, RM dysfunction was associated with the use of NIV during hospitalisation. Whilst the direct effect of SARS-CoV-2 on RM needs further research, these findings support the introduction of MIP/MEP measurements as screening for RM dysfunction at the post-COVID follow-up, regardless of symptoms, and the consideration of RM training [42] in patients identified as having moderate-to-severe dysfunction [25,43].

## Figures and Tables

**Table 1:** Sample characteristics and hospital admission factors of patients attending follow-up by MIP/MEP dysfunction.

	Overall *n* = 223 (100)	MIP/MEP Abnormal Values * *n* = 121 (54.2)	MIP/MEP Normal Values * *n* = 102 (45.8)	*p* Value
Demographics				
Age, years [IQR]	67 (57–75)	67 (59–74)	65 (55–75)	0.11
Male gender, no. [%]	153 (68.6)	76 (62.8)	77 (75.5)	0.04
Ethnicity				
Caucasian, n [%]	218 (97.8)	118 (97.5)	100 (98)	0.99
Black, n [%]	4 (1.8)	2 (1.7)	2 (2)	0.99
Asian, n [%]	1 (0.4)	1 (0.8)	0 (0)	0.99
Smoking history				
Current smoker, no. [%]	2 (0.9)	1 (0.8)	1 (1)	0.99
Former smoker, no. [%]	88 (39.5)	51 (42.1)	37 (36.2)	0.41
Non-smoker, no. [%]	133 (59.6)	69 (57)	64 (62.7)	0.41
BMI (pre-admission), median [IQR]	30 (27–34)	30 (27–34)	30 (27–34)	0.64
BMI > 30, no. [%]	121 (54.3)	68 (56.2)	53 (52)	0.21
Outcomes				
LOS, days [IQR]	14 (10–21)	15 (11–23.5)	13 (9–19.3)	0.14
Respiratory support				
HFNC, no. [%]	60 (26.9)	32 (26.4)	28 (27.5)	0.88
NIV, no. [%]	74 (33.2)	48 (39.7)	26 (25.5)	0.03
Intubation/MV, no. [%]	36 (16.1)	21 (17.4)	15 (14.7)	0.72
Use of Tocilizumab	150 (67.3)	79 (65.3)	71 (69.6)	0.47
Use of Dexamethasone	67 (30)	37 (16.6)	30 (13.4)	0.88
Use of other corticosteroids (oral/IV)	134 (60.1)	73 (60.3)	61 (59.8)	0.99
O_2_ at discharge, no. [%]	32 (14.3)	19 (15.7)	13 (12.75)	0.57
Follow up				
Dyspnoea grade (mMRC), median [IQR]	0 (0–1)	0 (0–1)	0 (0–1)	0.09
Dyspnoea as mMRC ≥ 1, no. [%]	67 (30)	39 (32.2)	28 (27.5)	0.46
FEV_1_/FVC, % [IQR]	82 (78–85.7)	81.8 (78.7–86.3)	82.1 (78.1–85.1)	0.78
TLC, %pred [IQR]	105 (94–116)	101 (91.5–113)	107.5 (96.8–117)	0.06
TLC < 90%pred, no. [%]	38 (17)	27 (22.3)	11 (10.8)	0.03
DLCO, %pred [IQR]	77 (67–87)	75 (64.5–86)	79.5 (68–92.3)	0.02
DLCO < 80%pred, no. [%]	128 (57.4)	77 (63.6)	51 (50.5)	0.06
MIP, %pred [IQR]	84 (66–104)	68 (58–73)	102 (90–120)	<0.0001
MEP, %pred [IQR]	82 (62–93)	65 (54–72)	96 (88–101)	<0.0001
Reduced strength in dominant hand, no. [%]	60 (26.9)	40 (33.1)	20 (19.6)	0.034
Reduced strength in right hand, no. [%]	68 (30.5)	46 (38)	22 (21.6)	0.01
Reduced strength in left hand, no. [%]	77 (34.5)	46 (38)	31 (30.4)	0.26

Abbreviations: BMI, body mass index; LOS, length of stay; mMRC, modified Medical Research Council; FEV_1_, forced expiratory volume in 1 s; FVC, forced vital capacity; TLC, total lung capacity; DLCO, diffusing capacity of the lungs for carbon monoxide; MIP, maximal inspiratory pressure; MEP, maximal expiratory pressure. * Lista-Paz A et al. Arch Bronconeumol 2023; 59(12):813–820 [13].

**Table 2:** Raw and independent association between clinical variables and MIP/MEP dysfunction at follow-up according to Lista-Paz A et al. 2023 [13].

	Univariable			Multivariable		
Parameter	OR	95% Confidence Interval	p Value	aOR	95% Confidence Interval	p Value
Age	1.02	0.99–1.04	0.12	1.01	0.95–1.06	0.09
Female sex	1.85	1.02–3.98	0.04	1.76	1.09–4.16	0.03 *
Smoking status (active/former)	1.19	0.91–1.55	0.20	1.11	0.89–1.57	0.29
BMI	1.01	0.96–1.07	0.64	1.09	0.97–1.08	0.66
BMI ≥ 30 kg/m^2^	1.19	0.7–2	0.53	1.21	0.78–1.99	0.51
Hospital length of stay	1.01	0.99–1.03	0.15	1.00	0.43–1.00	0.45
HFNC	0.95	0.52–1.93	0.87	0.91	0.99–1.76	0.61
NIV	1.79	1.01–3.22	0.04	1.91	1.07–3.49	0.04 *
Intubation/MV	1.22	0.59–2.54	0.59	1.31	0.61–2.78	0.43
Use of Dexamethasone	1.06	0.59–1.6	0.85	1.1	0.67–1.71	0.71
Use of other corticosteroids	1.02	0.6–1.75	0.94	1.01	0.52–1.65	0.97
Use of Tocilizumab	0.82	0.47–1.44	0.49	0.88	0.58–1.98	0.78

Abbreviations: OR, odds ratio; aOR, adjusted odds ratio; IQR, interquartile range; HFNC, high-flow nasal cannula oxygen therapy; NIV, non-invasive ventilation; MV, mechanical ventilation; BMI, body mass index. * *p* ≤ 0.05 statistically significant result.

## Data Availability

The dataset used and analysed during the study is available from the corresponding author on reasonable request and with ethical permission.
